# Nutritional status of in-school children and its associated factors in Denkyembour District, eastern region, Ghana: comparing schools with feeding and non-school feeding policies

**DOI:** 10.1186/s12937-018-0321-6

**Published:** 2018-01-12

**Authors:** Mavis Pearl Kwabla, Charlotte Gyan, Francis Zotor

**Affiliations:** 1 Department of Epidemiology and Biostatistics, School of Public Health, University of Health and Allied Sciences, Ho, Ghana; 2 Department of Family and Community Health, School of Public Health, University of Health and Allied Sciences, Ho, Ghana

**Keywords:** Malnutrition, School-age children, Stunting, Thinness, Overweight

## Abstract

**Background:**

Childhood malnutrition still remains a major public health problem impacting negatively on the academic aptitude of school-aged children (SAC) particularly in limited resource countries. The Government of Ghana in collaboration with the Dutch Government introduced the school feeding programme (SFP) to boost the nutritional status of SAC in the country. This study sought to compare the nutritional status of SAC enrolled in schools with the SFP and SAC enrolled in schools without the SFP in place for the purpose of identifying which group has the higher rate of malnutrition.

**Methods:**

A multi-stage sampling was used to select 359 SAC between 5 and 12 years who are enrolled in primary one to six. Twelve public schools were selected, of which 6 schools benefit from the SFP and the other six do not. Anthropometric measurements were conducted for the subjects and SPSS version 20.0 was used for data entry and analysis. Chi square test was carried out to determine the difference between the two groups of schools.

**Results:**

Of the total of 359 subjects, 55.1% were from schools that do not implement the SFP and 44.9% were from schools that implement the SFP. The prevalence of stunting among children in schools on the SFP was 16.2% compared with 17.2% among children in schools that do not implement the SFP. The prevalence of thinness was two times higher (9.3%) among children in schools on the SFP than in children in schools that do not implement the SFP (4.6%) (*p* = 0.028). The prevalence of overweight among children in schools on the SFP was 1.9% and 0.0% for children in schools that do not implement the SFP. Sub district, sex, age of pupil, area of residence and community type were significantly associated with stunting (*p* = 0.002), (*p* = 0.008), (p = 0.008), (*p* < 0.001) and (*p* = 0.007) respectively.

**Conclusion:**

Overweight and thinness were higher among children in schools on SFP than in children in schools without SFP. An evaluation of the implementation of the school feeding programme is recommended for future studies.

## Background

Good nutrition is the foundation to proper growth and development in every human, the absence of which individuals are subjected to multiple health complications. Malnutrition still remains a significant problem all over the world, especially among children [1] . Childhood malnutrition continues to be a public health problem of school-aged children in limited resource countries [2]. Of the total of 7.6 million deaths reported by the World Health Organization (WHO) in 2010, 64% were attributable to infectious causes [3] but their severity was greater when confounded by malnutrition especially in children who are unable to mount an effective immune response [4, 5]. In 2014, an estimated 95 million children under five years of age in less developed regions were underweight with the highest prevalence rates in Southern Asia (28%), followed by Western Africa (20%) [1]. In 2012 also, there was a recorded 6.6 million deaths among children below five years as a result of malnutrition [1]. Malnutrition leads to delay in physical growth and motor development, lower intelligent quotient, behavioural problems and poor social skills and an increase in the risk of morbidity and mortality [6].

Childhood malnutrition is triggered by multiple risk factors which when tackled can lead to a significant reduction in cases as seen in children. The common causes of malnutrition are frequent infections and lack of access to nutritious foods [1].

In response to the high incidents of diseases caused by malnutrition among children, interventions such as school-based nutrition education, deworming, food fortification, supplementation and school feeding programmes were introduced. [1]. Majority of these interventions have proven to be effective since their implementation. National-scale fortification for instance which includes the addition of micronutrients to staple foods in factories has proven to be an effective method to improve the health of populations [2]. The Ghana school feeding programme (GSFP) introduced in 2005 in some selected schools is one of such interventions to enhance nutrition and improve school attendance and education outcomes [7]. In resource limited nations, majority of school-aged children often walk long distances to school without having a morning and midday meal due to poverty [8]. The school feeding programme therefore offers these children with at least one free meal during school hours. However, because resources are generally limited in the poorest countries and providing food can be expensive, targeting communities that lack the resources to adequately provide for their school-age children is a critical element in improving the impact and penetration of school feeding programmes [9]. It was against this background that the school feeding programme in Ghana has not been scaled up to cover all schools in the country so that the programme can provide the greatest benefit to those most in need [9].

On a global scale, an estimated 60 million children attend school hungry every day with about 40% in Africa [9] whereas 65 million children suffer from chronic under nutrition globally due to inadequate food intake, frequent infection or both. Efforts by the World Food Programme (WFP) has helped in providing school meals to between 20 and 25 million children across 63 countries each year, often in the hardest-to-reach areas. Lunch programmes in schools have improved school enrolment, increased attendance, decreased dropout rates and raised nutrition levels among the children [2].

Following the implementation of the school feeding programme in Ghana, numerous challenges emerged including challenges of linking the program with local farmers, delay in the release of funds, inadequate resources for effective monitoring and inadequate training for caterers on health and nutrition related issues [10]. In addition, findings about the benefits of the SFP appear inconclusive. Whilst some studies found the SFP to impact positively on the nutritional status of SAC, others have not witness any significant difference in the nutritional status of children in schools on the feeding programme compared with those in schools that do not implement the school feeding programme [11, 12]. The GSFP has been in implementation for over 10 years now and as such, its impacts on the nutritional status of children can be well ascertained. Hence the study assessed the nutritional status of children in schools on the school feeding programme and those in schools that do not implement the feeding programme in Denkyembour District.

## Methods

### Study area

Denkyembour District is one of the 26 Administrative Districts in the Eastern Region of Ghana. It is located at the South-Western part of the Eastern Region and shares boundaries with Kwaebibirem and Akyemansa Districts to the North, West Akim Municipality to the South and Birim Central Municipality to the South-West.

The district has 78 Communities with an estimated population of 78,841 according to the Ghana 2010 population and housing census. It has a population growth rate of 2.3%. The district is a food and cash crop growing area with major cultivations being palm fruits and plantain. The district comprises of 6 sub districts and 23 public basic schools, of which 11 are on the school feeding programme and 12 do not implement the school feeding programme. The district was chosen based on the implementation of the school feeding programme in some selected public schools. This situation therefore provides the settings for evaluating the school feeding programme.

### Study population

This comprised of school children between ages 5 to 12 years who were in primary 1–6 and registered in the school for at least one academic year and whose parents or guardians provided consent for participation in the study. The children were interviewed together with their parents/teachers after which their anthropometric measurements were taken. Children with known health problems were excluded from the study.

### Study design

A cross-sectional study using multi-stage sampling was carried out from February to March 2016 to assess nutritional status of primary school-age children from 12 public schools in Denkyebour district of Ghana.

### Sample size determination

The sample size of 359 participants was determined using the formula:

$$ n=\frac{z^2\ast p(q)}{d^2} $$

Where, n is the desired sample size, z is the standard normal deviate, p is the estimated prevalence of stunting, q is 1.0 – p, and d is degree of accuracy desired. With the regional prevalence of stunting (17%), which is the highest among all the indicators, the calculation was based on stunting prevalence with confidence level of 95%.

Using a prevalence of stunting of 17% (DHS, 2015), z = 1.96, *p* = 0.28, q = 0.83 and d = 0.05 at a 95% confidence level,

$$ n=\frac{1.96^2(0.17)\ (0.83)}{0.05^2} $$

$$ n=217 $$

With the extra uncertainty about the true prevalence of stunting, design effect was considered in the sample size calculation. Therefore, the sample size was n*design effect (which is 1.5 in this case),

$$ n=217\times 1.5 $$

$$ n=326 $$

For a 10% non-response rate, the sample size was upwardly adjusted and rounded to 359 participants. This sample size was to ensure that, with probability of 95%, the estimated prevalence will fall within ±5% of the true population prevalence.

### Sampling

Respondents for the study comprised of school age children aged 5–12 years from two categories of Public schools (schools that implement the school feeding programme (SFP) and those that do not). They were selected from six stratified sub-districts using a multi-stage sampling technique. A list and population of all public schools on the SFP and those that do not implement SFP was obtained from the district education office following which 3 sub districts were purposively selected. For each sub district, two schools (one with the SFP and one without the SFP) were randomly selected. Proportionate sampling was used to determine the exact number of respondents to be recruited from each school. Respondents from the various schools were then selected based on balloting. A ballot involving the selection of a YES or NO was made for pupils in the selected schools who were in primary 1–6. Respondents who picked YES were the only ones interviewed together with their parents and or teachers.

### Data collection

After a pre-testing and training of research assistants, we used face-to-face interviews to administer the questionnaires to the pupils and or parents and teachers alike. Questionnaires administered to pupils solicited information about their personal characteristics (sex, age, area of residence, religion, and ethnicity) and dietary habits (number of meals intake per day and whether or not children receive money for food on school days) whereas that to the parents solicited information mainly about their occupation. Anthropometric measurements were taken using standard procedures. Weight and height readings were taken using a battery powered digital Seca 803 weighing scale (Medical measuring systems and scales, Hammer Steindamm 3–25, 22,089 Hamburg, Germany) and Seca 216 portable stadiometer (Medical measuring systems and scales, Hammer Steindamm 3–25, 22,089 Hamburg, Germany) respectively. Weight measurement was taken in duplicates and the average weight recorded. Before taking weight measurement, respondents were not permitted to wear shoes and heavy clothing except their school uniforms. Each school child was made to stand on the scale without holding onto any support with feet closed, hands by the sides and head in a forward position. Weight was then read immediately and recorded to the nearest 0.1 kg. Height was measured to the nearest 0.1 cm with a well mounted stadiometer. Before taking the measurements, respondents were asked to take off all foot wears and head gears or hats if any. Each pupil was made to stand with back against a wall, heels together and in line with the buttocks, shoulders and head. With a horizontal line of sight to the respondent, the head piece of the stadiometer was used to ensure that the top of the head was measured at right angle to the wall.

### Data analysis

Data was entered twice and checked using SPSS version 20.0. Chi square test was used for categorical variables to establish the differences between the 2 groups (schools with the school feeding programme and those without the school feeding programme). Multiple logistic regression analysis was used to confirm associations between dependent (nutritional status) and the independent variables (age, sex, mother’s occupation and community type).

## Results

A total of 359 school aged children (SAC) from 6 sub-districts were recruited for the study of which 55.2% were from schools that do not implement the school feeding programme and 44.9% were from schools on the school feeding programme. The mean age of the school aged children was 9.8 ± 1.9 years. It was found that most of the participants (69.4%) were from the rural communities with 72.1% staying with their biological parents. Majority (56.3%) of the participants were in the age category of 5–9 years with almost equal proportion of both sexes. Background and demographic data of participants is shown in Table 1 below.

**Table 1:** Background and demographic characteristics of participants

Mean age ± SD =9.8 ± 1.9		
Attribute	Frequency	Percent
	(*N* = 359)	
Sub-district		
Akwatia	97	27.0
Topremang	121	33.7
Okumaning	141	39.3
Sex of pupil		
Male	171	47.6
Female	188	52.4
Age of pupil(years)		
5–9	202	56.3
10–12	157	43.7
Area of residence		
Rural	249	69.4
Urban	110	30.6
Community type		
Mining	126	35.1
Farming	233	64.9
Religion		
Christian	327	91.1
Islam	31	8.6
Ethnicity		
Akan	238	66.3
Ewe	58	25.3
Ga	9	2.5
Northerners	24	6.7
Others	30	8.4
Child lives with:		
Both parents	259	72.1
Mother alone	43	12.0
Father alone	13	3.6
Other relations	44	12.3
Guardian’s Occupation		
Father’s occupation		
Farming	129	35.9
Mining	45	12.5
Civil/public servant	34	9.5
trading	19	5.3
Others	74	20.6
Unemployed	28	7.8
Mother’s occupation		
Farming	69	19.2
Mining	29	8.1
Civil/public servant	18	5.0
trading	189	52.7
Others	26	7.2
Unemployed	28	7.8
Receive money for school		
Yes	332	92.5
No	26	7.2
Frequency of meal intake per day		
< 1	0	0.0
> 2	359	100
Amount of money(Cedis) for school		
< 1.00	89	24.8
1.00–1.99	240	66.9
2.00–3.00	6	1.7
no money	24	6.7
Feeding type		
School feeding	161	44.9
Non-school feeding	198	55.2

### Dietary history of the participants

All participants in both schools on the school feeding programme and those in schools without the school feeding programme eat between 2 and 3 times (including snacks) in a day. Majority, 348 (96.9) frequently eat 3 times in a day whilst the rest eat 2 times in a day. None was reported to have taken less than one meal in a day. Of the 3 meals taken in a day, breakfast 269 (74.9) and lunch 78 (21.7) were normally taken at school whilst supper 232 (64.5) was taken at home. About 94% of pupils were given money to purchase food at school.

### Prevalence of stunting, thinness and overweight among the participants

Approximately, 16.7, 6.7 and 0.8% of all the respondents were stunted (HAZ), thin (WHZ) and overweight (BAZ) respectively and 0.6% found to be of tall stature or well nourished (Table 2).

**Table 2:** Nutritional status of the pupils

Attribute	All	
	Frequency	Percentage (%)
Height for age z-score (HAZ)		
Normal	297	82.7
Tall	2	0.6
Stunted	60	16.7
Total	359	(100.0)
Body mass index for age z-score (BAZ)		
Normal	332	92.5
Overweight	3	0.8
Thinness	24	6.7
Total	359	(100.0)

### Comparison of the levels of stunting, thinness, and overweight among study participants

The Prevalence of stunting was found to be higher among children in schools without the school feeding programme (17.2%) than in children in schools on the feeding programme (16.2%) but there was no significant difference (*p* = 0.284). The prevalence of thinness was about twice higher (9.3%) in children with the school feeding programme than in those without the programme (4.6%), (*p* = 0.028). Unfortunately, 1.9% of children in schools with the feeding programme were found to be overweight. Summary of the comparison can be found in Table 3 below.

**Table 3:** Nutritional status (comparison of school feeding and non- school feeding)

Attribute	All	School feeding	Non- school feeding	*p*-value
	Frequency (%)	Frequency (%)	Frequency (%)	
Height for age z-score(HAZ)				
Normal	297 (82.7)	133 (82.6)	164 (82.8)	
Tall	2 (0.6)	2 (1.2)	0 (0.0)	
Stunted	60 (16.7)	26 (16.2)	34 (17.2)	
Total	359 (100.0)	16 1(100.0)	198 (100)	0.284
Body mass index for age z-score(BAZ)				
Normal	332 (92.5)	143(88.8)	189 (95.5)	
Overweigh0074	3 (0.8)	3 (1.9)	0 (0.0)	
Thinness	24 (6.7)	15 (9.3)	9 (4.6)	
Total	359 (100.0)	161 (100.0)	198 (100.0)	0.028

### Risk factors associated with malnutrition among school age children

The study explored possible risk factors associated with malnutrition in school age children. Overweight, thinness and stunting were the outcome variables measured. In exploring the possible risk factors associated with malnutrition, the study reveals that occupation of mother affects the overweight status of the child, a chi squared test performed at a significant level of 0.05, revealed a strong significant association between the two variables (χ^2^ = 15.37, *p* = 0.009, α = 5%).

With regards to thinness, sex and age were proven to be significantly associated with thinness with a chi square and *p*-value of (χ^2^ = 5.55, *p* = 0.018, α = 5%) and (χ^2^ = 5.48, *p* = 0.019, α = 5%) respectively, however a logistic regression performed to test the strength of the association indicated that female is a strong indicator of thinness as females are 2.7 times likely to become thin as compared to males [OR = 2.7 (95% CI = 1.70–6.70), *p* = 0.023].

With respect to stunting, Sub district, sex of pupil, age of pupil, area of residence and community type were significantly associated with stunting at bivariate analysis level, with a chi square values and *p*-values of (χ^2^ = 12.29, *p* = 0.002, α = 5%), (χ^2^ = 7.12, *p* = 0.008, α = 5%), (χ^2^ = 6.94, p = 0.008, α = 5%), (χ^2^ = 12.20, *p* < 0.001, α = 5%) and (χ^2^ = 7.21, *p* = 0.007, α = 5%) respectively.

However logistic regression revealed that only sub district and sex of pupils were strong indicators of stunting with odds ratio of [OR = 1.7 (95% CI = 0.16–18.60), *p* = 0.001] and [OR = 2.0 (95% CI = 1.12–3.67), p = 0.007] respectively. Refer Table 4 below.

**Table 4:** Factors associated with malnutrition among school age children

Attribute	N (%)	N (%)	Pearson chi-square		AOR (95% CI)
			X^2^	*P*-value	
Overweight status					
Occupation of mother	Not Overweight	Overweight			
Mining	27 (93.1)	2 (6.9)	15.37	0.009	0.637
Farming	68 (98.6)	1 (1.4)			
Trading	189 (100.0)	0 (0.0)			
Civil/public servant	18 (100.0)	0 (0.0)			
Other	26 (100.0)	0 (0.0)			
Unemployed	28 (100.0)	0 (0.0)			
Thinness					
sex	Not Thin	Thin			
Males	181 (96.3)	7 (3.7)	5.55	0.018	1
Female	154 (90.1)	17 (9.9)			2.7 (1.08–6.70)
Age (years)					
5–9	183 (90.6)	19 (9.4)	5.48	0.019	1
10–12	152 (96.8)	5 (3.2)			0.3 (0.12–0.93)
Stunting					
Sub-district	Not stunted	Stunted			
Akwatia	91 (93.8)	6 (6.2)	12.29	0.002	1
Topremang	100 (82.6)	21 (17.4)			1.7 (0.16–18.60)
Okumaning	108 (76.6)	33 (23.4)			
sex of pupil					
Male	166 (88.3)	22 (11.7)	7.12	0.008	1
Female	133 (77.8)	38 (22.2)			2.0 (1.12–3.67)
Age of pupil (years)					
5–9	159 (78.7)	43 (21.3)	6.94	0.008	1
10–12	140 (89.2)	17 (10.8)			0.5 (0.27–0.96)
Area of residence					
Rural	196 (78.7)	53 (21.3)	12.20	<0.001	1
Urban	103 (93.6)	7 (6.4)			0.4 (0.05–3.20)
Community type					
Mining	185 (79.4)	48 (20.6)	7.21	0.007	1
Farming	114 (90.5)	12 (9.5)			0.n (0.36–2.73)

Chi-square test, significance defined as *p* < 0.05. Stunting defined as HAZ < −2 Wasting defined as WHZ < −2 and Overweight/Obesity defined as BMI-for-Age > +1SD

## Discussion

The study sought to compare nutritional status of school age children (SAC) enrolled in schools benefiting from the school feeding programme and those that do not for the purpose of identifying which group has the higher rate of malnutrition. Anthropometric measurements of respondents were taken to evaluate the health and nutritional status of the children and the indices that directly reflect the socio-economic status of the family, health and social wellbeing of the population. Overweight, thinness and stunting were the anthropometric indicators used to measure malnutrition in the children.

Our study found an over-all prevalence of 0.8% for overweight among the school children in the Denkyembour District. This prevalence is lower compared to what was found in other studies [2, 12–14]. On the contrary, as high as 67% of children in schools on school feeding programme in the La-Nkwatanang Madina District were found to be stunted, underweight and anaemic [15]. The differences observed may be attributed to geographical differences: participants in this current study were from a rural setting where majority of the populace were farmers and may tend to consume more energy dense foods whereas La-Nkwatanang is a peri-urban area. The different makes of weighing instruments used in the measurement of weight and the different methods used in the classification of weight status by the different authors may also play a role.

The nutritional status of school aged children in the two genre of schools recruited for this study revealed an overweight prevalence of 1.9% among pupils in schools on the school feeding programme compared to 0.0% for those in schools without the feeding programme. Surprisingly, prevalence of thinness was also found to be higher among children in schools with the feeding programme (9.3%) than in those in schools without the feeding programme (4.6%) which concur with result of a similar study done in the Nkwanta South District [16]. On the contrary, a study conducted in Kenya [17] reported that Children participating in the school feeding programme were less wasted than children in the control group (*P* = 0.002).

The higher prevalence of overweight among children in schools with the school feeding programme compared to those in schools without the programme found in this study could be due to parents’ lack of adequate knowledge on good nutrition practices to provide their children with healthy foods. This could also be as a result of the nutritional transition characterised by consumption of a more energy and high fat diet coupled with low physical activity in such schools. Furthermore, a more worrying findings in this study was the high prevalence of thinness found among children in schools on school feeding programme as compared with children in schools without school feeding programmes since it is expected that children in feeding programme receive supplementary food intended to improve their nutrition. This result confirms the findings of a study where the food being served by the school feeding programme was found not to meet the recommended energy from macronutrient intake as set by World Food Programme [18]. This could be as a result of the high level of poverty in the communities selected for the feeding programme since one of the aims of the feeding programme was to target communities that lack adequate resources to provide for their school age children. A report by the GSFP also indicated that the feeding programme is faced with numerous operational challenges including inconsistent release of funds making feeding irregular and the food not meeting the recommended energy and micronutrient levels [9, 10, 19] and this could also be a contributing factor.

The study found an association between overweight and parent’s occupation (*p* = 0.009) which is in conformity with a similar study (13), which reported a significant association between overweight/obesity and father’s occupation (<0.05). Their study also found overweight and obesity to be more prevalent in children belonging to affluent and higher socioeconomic group or families. This could mean that children from affluent homes have more fat and protein components in their diet with neglect to physical activity. Excessive consumption of calories from carbohydrate intake by these children could play a role in their overweight status. Consequently, their energy intake is more than energy expenditure, hence the accumulation of fat.

### Limitation

Cross-sectional design used in the study was not appropriate to establish a causal relationship between provision of school meals and nutritional status (stunting, thinness, and underweight). We could not also estimate the effect of challenges facing the feeding programme such as missed and irregular meals. Impact assessment was not also undertaken as a result of the unavailability of baseline information about the nutrition status of the children at the start of the feeding program.

## Conclusion

The overall prevalence of overweight was low compared to that found in other studies. However, prevalence rates of overweight and thinness were found to be higher among children in schools on the school feeding programme compared to children in schools without the feeding programme. An evaluation of the implementation of the school feeding programme is recommended for future studies.

## Abbreviations

GSFP: Ghana school feeding programme
SAC: School age children
SFP: School feeding programme
WFP: World food programme

## References

[1] M Do, Monteiro C, Akré J, Clugston G. Global Database on Child Growth and Malnutrition The worldwide magnitude of protein ­ energy malnutrition : an overview from the WHO Global Database on Child Growth. Bull World Health Organ. 2015; 2015. PMC2393544 8313488

[2] Lardner D, Giordano J, Jung MK, Passafaro MD, Small A, Haar M (2015). Evaluation of nutritional status among school-aged children in rural Kwahu-Eastern region, Ghana; anthropometric measures and environmental influences. AJFANDce.

[3] Liu L, Johnson HL, Cousens S, Perin J, Scott S, Lawn JE (2012). Global, regional, and national causes of child mortality: an updated systematic analysis for 2010 with time trends since 2000. Lancet.

[4] Rice AL, Sacco L, Hyder A, Black RE (2000). Malnutrition as an underlying cause of childhood deaths associated with infectious diseases in developing countries. Bull World Health Organ.

[5] Rayhan MI, Khan MSH (2006). Factors causing malnutrition among under five children in Bangladesh. Pak J Nutr.

[6] UNICEF (2013). Improving child nutrition: The achievable imperative for global progress. Division of Communication UNICEF.

[7] Gelli A, Masset E, Folson G, Kusi A, Arhinful DK, Asante F (2016). Evaluation of alternative school feeding models on nutrition, education, agriculture and other social outcomes in Ghana: rationale, randomised design and baseline data. Trials.

[8] Intiful FD, Lartey A (2014). Breakfast habits among school children in selected communities in the eastern region of Ghana. Ghana Med J.

[9] Buhl A (2010). Meeting Nutritional Needs Through School Feeding: A Snapshot of Four African Nations.

[10] WHO (2006). The world health report 2006: working together for health. World Health.

[11] Quaye W, Essegbey G, Frempong G, Ruivenkamp G (2010). Understanding the concept of food sovereignty using the Ghana school feeding Programme (GSFP). Int Rev Sociol.

[12] Danquah AO, Amoah AN, Steiner-Asiedu M, Opare-Obisaw C (2012). Nutritional Status of Participating and Non-participating Pupils in the Ghana School Feeding Programme. J Food Res.

[13] Vohra R, Bhardwaj P, Srivastava JP, Srivastava S, Vohra A (2011). Overweight and obesity among school-going children of Lucknow city. J Fam Community Med.

[14] Ghana Statistical Service (2014). Ghana Demographic and Health Survey 2014: Ghana statistical service, Ghana Health Service. Ghana Statistical Service (GSS) Ghana Demographic and Health Survey.

[15] Owusu JS, Colecraft EK, Aryeetey R, Vaccaro JA, Huffman FG. Nutrition Intakes and Nutritional Status of School Age Children in Ghana. J Food Res. 2017;6(2) Available from: 10.5539/jfr.v6n2p11

[16] Prince A, Laar A. Nutritional Status of School-Age Children in the Nkwanta South District -Volta Region of Ghana. Eur Scientif J. 2014, 1010:1857–7881. Available from: http://www.eujournal.org/index.php/esj/article/view/4462/4272.

[17] Neervoort F, von Rosenstiel I, Bongers K, Demetriades M, Shacola M, Wolffers I (2013). Effect of a school feeding programme on nutritional status and anaemia in an urban slum: a preliminary evaluation in Kenya. J Trop Pediatr.

